# Inadvertent Intrathecal Administration of Digoxin, with Review of the Literature

**DOI:** 10.1155/2023/4034919

**Published:** 2023-10-20

**Authors:** Burton J. Tabaac, Ian O. T. Laughrey, Hany F. Ghali

**Affiliations:** ^1^University of Nevada, Reno, School of Medicine, Reno, NV, USA; ^2^Department of Neurology, Carson Tahoe Health, Carson City, NV, USA; ^3^Department of Intensive Care, Carson Tahoe Health, Carson City, NV, USA

## Abstract

While the systemic effects of digoxin have been studied, limited data exist on the effects of neuraxial administration. Prior case reports document how digoxin and lidocaine share indistinguishable vials and were inadvertently stocked together in spinal and epidural anesthesia kits, necessitating a need for further implementation of safety measures. Here, we report the poor progression and brain death of a postpartum woman after accidental administration of intrathecal digoxin during a routine elective cesarean section (C-section). It is imperative that quality improvement and safety measures are taken to avoid the recurrence of this medical error.

## 1. Introduction

There are several case reports on neuraxial digoxin administration with spontaneous resolution of sequelae after minimal medical intervention [1, 2]. To our knowledge, this is the first death reported. We report the details of the accidental administration of intrathecal digoxin during an epidural for a cesarean section (C-section) of a young woman, including the medical management, encephalopathic changes, diagnostic findings, and ultimate withdrawal of supportive care. Given that this has occurred in the past, we highlight the need for the implementation of safety regulations. We also share our hypotheses for the patient's anoxic brain injury. Written consent has been obtained from the patient's durable power of attorney for the publication of this case report.

## 2. Case Presentation

A 34-year-old woman, G1P0 at 37 weeks, with placenta previa and a past medical history of anxiety and hypothyroidism, was admitted for an elective C-section. Spinal anesthesia was reportedly administered at 8:05 AM (2 ml bupivacaine 0.75% and 25 mg fentanyl intrathecally with 0.1 mg morphine sulfate intravenously) after which the patient underwent epidural anesthesia with an additional dose of bupivacaine due to lack of anticipated response from the first dose (the dose was not documented) at 9:14 AM. The anesthetist did not scan the barcode or read the label aloud to another staff member prior to administration. C-section was completed successfully at 9:56 AM. Neurologic examination both prior to and during C-section was normal. Intraoperatively, the patient reported a cough, nausea, and vomiting. Postoperatively, the patient complained of lightheadedness, fatigue, and blurred vision. Vital signs immediately postoperative were as follows: blood pressure: 111 mmHg systolic and 66 mmHg diastolic, temperature: 35.9°C (96.6 F), heart rate: 77 beats per minute, and respiratory rate (RR): 20 breaths per minute. En route to the labor and delivery postanesthesia care unit (PACU), at 10:18 AM, the patient was reported to have hypoxemia requiring increased oxygen supplementation. The patient was noted to be completely paralyzed at this time with decreased responsiveness and apnea. Vital signs were captured as follows: blood pressure: 121 mmHg systolic and 76 mmHg diastolic, heart rate: 100 beats per minute, temperature: 36.4°C (97.5 F), and RR: 18 breaths per minute. The patient was transferred to the intensive care unit and treated with intravenous (IV) flumazenil to reverse 2 mg IV midazolam administered by anesthesiologists. Given deterioration, a rapid response team was called, and the patient was intubated by the rapid response team. The rapid response team noted the patient to be unresponsive to noxious stimuli, with dilated fixed pupils, and absent gag, cough, and corneal reflexes. Head and chest computed tomography (CT) scans at that time revealed no acute findings.

Given the concern for possible administration of an unreported neuromuscular paralytic, sugammadex IV was administered by the intensivist, with no change in status. During chart review, around 5:00 PM, the anesthesiology team discovered the patient had inadvertently received intrathecal digoxin instead of planned bupivacaine during spinal anesthesia. Serum digoxin levels were low on serial testing (0.7 ng/ml and 0.6 ng/ml measured 6 hours apart). The patient was managed on low-dose propofol infusion due to concern for potential wakefulness while paralyzed.

On day 2 of hospital admission, propofol was discontinued to aid in assessment of mental status, scoring a 4 on train-of-four (TOF) response in the facial muscles. The patient remained unresponsive, with eight spontaneous shallow breaths per minute, absent brainstem reflexes, and no response to noxious stimuli.

An electroencephalogram revealed no seizure activity but did show evidence of diffuse encephalopathy. The patient was treated with IV methylprednisolone (1 gram daily for 3 days) with an empiric IV infusion of 18 vials of digoxin-specific antibody (DIGIFab) (40 mg per vial). Since the exact amount of digoxin administered was unknown, the manufacturer's dosing for acute ingestion of unknown amounts of digoxin was utilized.

**Figure 1:**
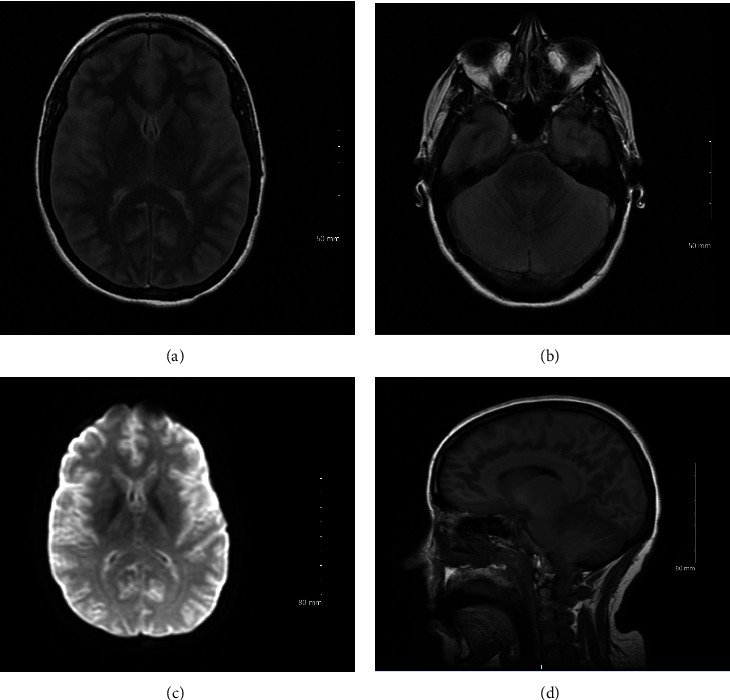
(a) MRI brain, axial T2 FLAIR, at the level of the ganglionic layer. (b) MRI brain, axial T2 FLAIR, at the level of the 4th ventricle. (c) MRI brain, axial DWI sequence. (d) MRI brain, sagittal T1 sequence.

**Figure 2:**
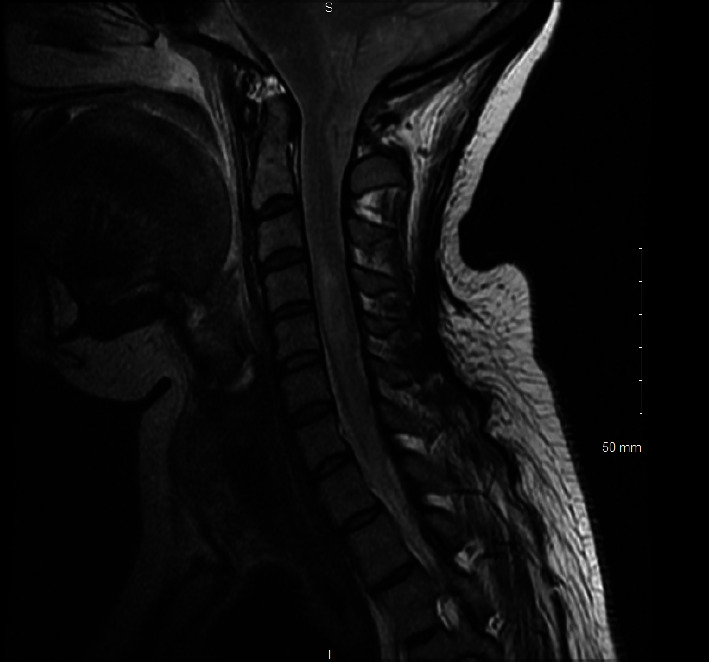
MRI C-spine, sagittal T2 sequence.

**Figure 3:**
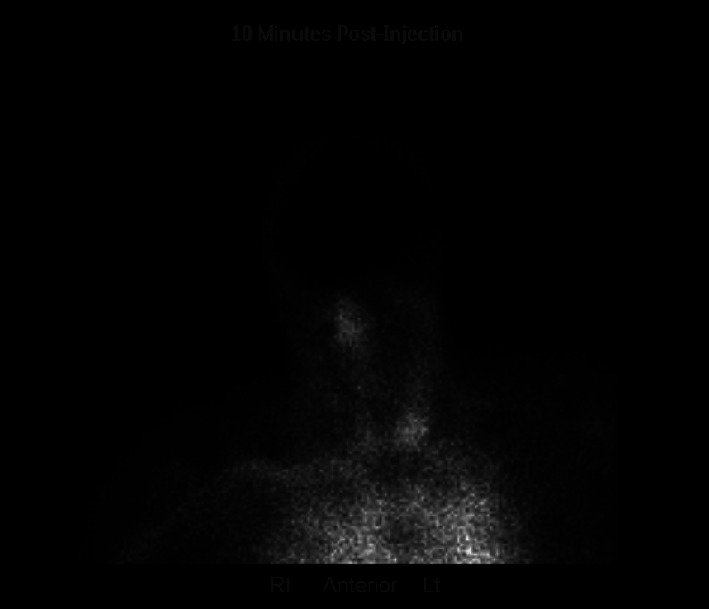
Nuclear medicine brain scan.

On day 3, sedation medications were held without any improvement and severe anoxic brain injury was suspected. Brain magnetic resonance imaging (MRI) (Figure 1) revealed severe cerebral edema with mild tonsillar herniation. Cervical spine MRI (Figure 2) revealed diffuse edema. A nuclear medicine cerebral blood flow study (Figure 3) revealed no intracranial activity, consistent with brain death. After a discussion with the patient's family, life support was withdrawn in keeping with their wishes and goals of care.

## 3. Discussion

In similarly reported cases in the literature, all patients presented with motor and sensory deficits, while some also had an altered level of consciousness and/or diminished brainstem reflexes [1–3]. In prior cases, the overall prognosis was favorable with the resolution of neurological deficits or residual weakness upon discharge [1–3]. The common strategy in reviewed cases was supportive care until resolution of the symptoms, which ranged from 2 to 7 days [1–3].  Table 1 delineates cases with surgical procedures and outcomes; this demonstrates reported cases of intrathecal tranexamic acid, where those related to caesarean birth appeared to have higher mortality, underscoring the hypothesis that pregnancy is a risk factor for adverse outcomes from spinal drug errors.

Systemic administration of digoxin has been well studied with the mechanism of action for digoxin on cardiac cells being understood as a reversible sodium/potassium ATPase inhibitor [4]. Inhibition of the sodium/potassium ATPase pump increases intracellular sodium in cardiac myocytes, increasing contractility. There are little clinical data on the effects of digoxin on the central nervous system.

One published animal study on rabbits demonstrates spinal blockade via intrathecal administration of digoxin, resulting in cardiopulmonary arrest and death [5]. Of the six similar cases reported, three described spontaneous resolution within 24 hours [1], one case with clinical resolution within two days after administration of high-dose steroids [2], and one case with resolution after one week of intracranial pressure management, IV DIGIFab administration, intra-arterial verapamil delivered, and administration of IV milrinone [3]. One documented case also addressed via intracranial pressure management, IV DIGIFab administration, intra-arterial verapamil, and IV milrinone yet did not result in improvement in lower extremity paraplegia [3].

Diagnostic findings of our case are consistent with severe anoxic brain injury. In comparing this patient to previously reported cases, it is important to emphasize that only one patient had experienced severe encephalopathic changes with similar diagnostic findings of anoxic brain injury [3]. In both cases, intrathecal digoxin was inadvertently administered in lieu of epidural anesthetic during a routine C-section, with both patients requiring intubation [3].

Although the pathogenesis of the patient's anoxic brain injury after digoxin is unclear, we hypothesize several possibilities. First, the anoxic brain injury may be a direct sequela of intrathecal digoxin. There is evidence that neurons possess three isoforms of the sodium/potassium ATPase pump (*⍺*1, *⍺*2, and *⍺*3) [6]. Binding of digoxin to these isoforms can result in increased intracellular sodium leading to cerebral edema. Of the six prior case reports, four did not document imaging results with MRI sequencing, and it is unknown if cerebral edema was present in those cases [1, 2]. The cases for which intrathecal digoxin was administered without a severe outcome when compared to our patient suggest other factors at play that can place patients at risk for a poor prognosis [1, 2].

Another hypothesis for anoxic brain injury is the possibility of cerebral ischemia occurring prior to intubation. The admitting team maintained the impression that our patient had minimal apnea prior to intubation, yet a thorough review of the medical records revealed it is unclear how long the patient experienced postoperative apnea. If apnea was prolonged, it may have been the cause for the anoxic brain injury.

In correspondence with authors of two case reports that documented patients who had undergone intrathecal digoxin administration during C-sections, it was noted that their patients presented with cerebral vasospasm [3]. This is an important neurological sequela to consider as vasospasm can be a potential cause of anoxic brain injury [3]. All documented cases of peripartum intrathecal digoxin administration (including this report) resulted in cerebral and spinal edema, with pregnancy playing an unclear role in neurological sequelae severity [3]. In review of the literature, there is another case documented in which drug errors with intrathecal tranexamic acid occurred during cesarean delivery with catastrophic results [5].

**Figure 4:**
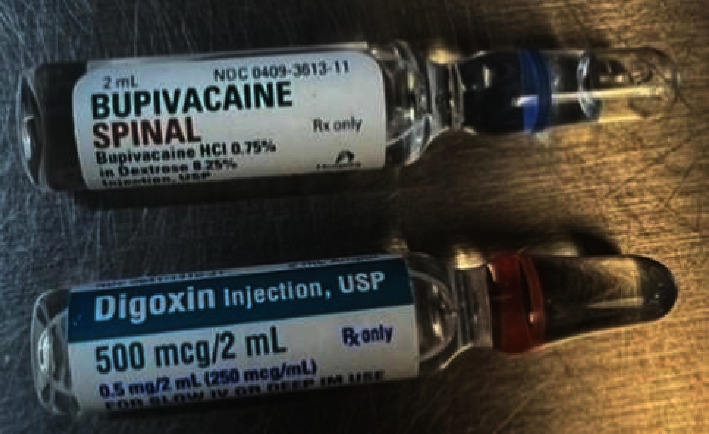
Vials for digoxin and bupivacaine. Bupivacaine spinal by Hospira (NDC 0409-3613-11) and digoxin by Hikma Pharmaceuticals (NDC 0641-1410-31).

A contributor to the root cause of this error in documented cases is similarity in appearance between the vials of digoxin and local anesthetics as depicted in Figure 4 [1]. This contributed to the same error in two nonrelated medical institutions, suggesting a systemic risk of stocking similar appearing medications inappropriately.

## 4. Conclusion

Inadvertent intrathecal injection of digoxin is a rare occurrence, yet it has been reported on six other occasions in the published literature to date. Drug errors may be missed unless someone thinks of this possibility and is able to discover the error. Thus, the incidence may be greater than reports suggest. This preventable medication error resulted in an untimely death of a young mother, leaving the family to grieve over the loss of their loved one. We suggest the consideration of quality control measures. This may include standardizing a redesign of the labeling of medications that share similar vials and exist in similar workspaces, implementing standardized inventory training of medical staff who stock medication kits, reevaluating safety training of medication administration, and creating a panel of medical professionals at hospital institutions to remain up-to-date on medical errors as this will promote knowledge sharing of potential management when medication errors occur.

If this error were to recur, we strongly recommend obtaining early brain and spine MRI imaging in conjunction with a rapid assessment of intracranial pressure and cerebral perfusion, with empiric treatment targeting control of intracranial pressure and vasospasms, paired with standard supportive care measures. It is relevant to underscore that some patients may require up to 7 days to demonstrate recovery given the half-life of digoxin.

## Figures and Tables

**Table 1:** Cases with surgical procedure details and outcomes with intrathecal digoxin.

Case, age, gender	Medical history	Procedure	Type of block	Amount of digoxin administered intrathecally	Physical exam findings	Diagnostic findings	Treatment	Number of days to recovery
Case 1: 21 y.o. male [1]	ASA physical status 1 [1]	Thrombotic hemorrhoidectomy [1]	Spinal anesthesia [1]	0.5 mg [1]	Paresthesias and paralysis for lower extremities to level of umbilicus, absent lower limb reflexes [1]	Normal: chest X-ray, electroencephalogram, electrocardiogram, head CT [1]	Not reported [1]	Full recovery at post-op 1 day (24 hours). Discharged on post-op day 2, no long-term sequelae at 5 month follow-up [1]

Case 2: not reported [1]	Not reported [1]	Not reported, but noted to be similar to case 1 [1]	Not reported, but noted to be similar to case 1 [1]	0.5 mg [1]	Not reported, but noted to be similar to case 1 [1]	Not reported, but noted to be similar to case 1 [1]	Not reported [1]	Not reported, but noted to be similar to case 1 [1]

Case 3: not reported [1]	Not reported [1]	Not reported, but noted to be similar to case 1 [1]	Not reported, but noted to be similar to case 1 [1]	0.5 mg [1]	Not reported, but noted to be similar to case 1 [1]	Not reported, but noted to be similar to case 1 [1]	Not reported [1]	Not reported, but noted to be similar to case 1 [1]

Case 4: 52 y.o. male [2]	Complicated medical history significant for end-stage renal disease on hemodialysis three times a week [2]	Elective total hip hemiarthroplasty [2]	Spinal anesthesia [2]	0.4 mg [2]	Symmetric, bilateral lower extremity weakness with subsequent paraplegia and upper extremity heaviness, with consequent sensory deficits to the C4-C5 levels, agitation and confusion 6 hours after steroid drip, dyspnea, right-sided gaze, lack of orientation to time, person, or place [2]	Cervical, thoracic, and lumbar MRI as well as brain CT were negative for spinal hematoma or cord compression, potassium elevated at 6.4 mEq/L (5.6 mEq/L prior to surgery and 5.2 mEq/L at baseline), digoxin level 1.9 ng/mL, ECG showed no acute changes, ABG: 7.35/42/83/23.2 on 2 L nasal cannula, SpO_2_ 100%, repeat brain CT showed no acute changes, EEG showed no seizure activity [2]	Methylprednisolone 2500 mg IV, methylprednisolone drip at 5.4 mg/hr for 24 hours, calcium gluconate and regular insulin with 1 amp of D50W for hyperkalemia, with urgent hemodialysis, 2 L O_2_ nasal cannula for dyspnea [2]	Post-op day 2, the patient was alert and oriented to person and place, on evening of post-op day 3, the patient was able to move all extremities and follow simple commands

Case 5: female, age not reported [3]	Peripartum, no other medical history reported [3]	Cesarean section [3]	Spinal anesthesia [3]	Not reported [3]	Decreased mental status followed by seizure, paraplegia of the limb [3]	Brain CT negative for acute pathology, MRI brain with significant bilateral frontal and temporal restriction with cortical ribboning, spinal MRI showed central cord edema with T2 signal changes, LP revealed high-opening pressure with elevated white blood cell counts and protein and low glucose, repeat MRI of the brain was normal and spine MRI with resolution of edema of the cervical spinal cord but persistent thoracic patchy signal intensity [3]	Intubation and ventilation, DIGIfab IV noted in report [3], additional treatment recommendations given by author via ResearchGate direct message: electroencephalography, trail antiepileptic drugs, external ventricular drain for shunting of cerebrovascular fluid, sustained low-efficiency dialysis to clear residual medication, CTA and MRA with DSA to evaluate for vasospasms with permissive HTN if pt is having vasospasms, intra-arterial verapamil for vasospasm, and IV milrinone	Approximately 1 week for recovery to baseline per direct discussion with the author of the report

Case 6: female, age not reported [3]	Peripartum, no other medical history reported [3]	Cesarean section [3]	Spinal anesthesia [3]	Not reported [3]	Decreased mental status followed by seizure, paraplegia of limbs [3]	Brain CT negative for acute pathology, MRI brain with significant bilateral frontal and temporal restriction with cortical ribboning, spinal MRI showed central cord edema with T2 signal changes, LP revealed high-opening pressure with elevated white blood cell counts and protein and low glucose, repeat MRI of the brain was normal and spine MRI with resolution of edema of the cervical spinal cord but persistent thoracic patchy signal intensity [3]	Intubation and ventilation, DIGIfab IV noted in report [3], additional treatment recommendations given by author via ResearchGate direct message: electroencephalography, trail antiepileptic drugs, external ventricular drain for shunting of cerebrovascular fluid, sustained low-efficiency dialysis to clear residual medication, CTA and MRA with DSA to evaluate for vasospasms with permissive HTN if pt is having vasospasms, intra-arterial verapamil for vasospasm, and IV milrinone	Approximately 1 week for recovery to baseline per direct discussion with the author of the report

Case 7 (our patient): 34 y.o. female	Peripartum, anxiety, hypothyroidism	Cesarean section	Spinal anesthesia	Not recorded in the patient's record. Given the volume of the draw, suspect 0.5 mg	Post-op day 1: lightheadedness, fatigue, blurred vision, hypoxemia, quadriplegia, altered mental status, decreased responsiveness, unresponsive to noxious stimuli, dilated fixed pupils, absent gag, cough, and corneal reflexes, apnea. On day 2 of hospital admission, the patient remained unresponsive, with eight spontaneous shallow breaths per minute, absent brainstem reflexes, and no response to noxious stimuli	Chest and head CT negative for acute findings, digoxin levels on serial testing: 0.7 ng/ml and 0.6 ng/ml measured 6 hours apart. On day 3, brain MRI revealed severe cerebral edema with mild tonsillar herniation. Cervical spine MRI revealed diffuse edema. A nuclear medicine cerebral blood flow study revealed no intracranial activity, consistent with brain death	Intubation and ventilation, sugammadex IV initially given prior to revelation of medication error, low-dose propofol infusion, IV methylprednisolone (1 gram daily for 3 days), empiric IV infusion of 18 vials of digoxin-specific antibody (DIGIFab) (40 mg per vial),	Withdrawal of life support and time of death reported on day 3 of hospitalization

## Abbreviations

C-section: Cesarean section
RR: Respiratory rate
IRB: Institutional Review Board
PACU: Postanesthesia care unit
CT: Computed tomography
IV: Intravenous
TOF: Train-of-four
DIGIFab: Digoxin-specific antibody
MRI: Magnetic resonance imaging
CSF: Cerebrospinal fluid.
